# 6-Thioguanine and Its Analogs Promote Apoptosis of Castration-Resistant Prostate Cancer Cells in a BRCA2-Dependent Manner

**DOI:** 10.3390/cancers11070945

**Published:** 2019-07-05

**Authors:** Luna Laera, Nicoletta Guaragnella, Sergio Giannattasio, Loredana Moro

**Affiliations:** 1 Institute of Biomembranes, Bioenergetics and Molecular Biotechnologies, National Research Council, Via Amendola 122/O, 70126 Bari, Italy; 2 Department of Biosciences, Biotechnology and Biopharmaceutics, University of Bari, Via Orabona 4, 70125 Bari, Italy

**Keywords:** prostate cancer, 6-thioguanine, paclitaxel, PARP inhibitors, BRCA2, apoptosis, yeast

## Abstract

*Background*: Mutations in the oncosuppressor gene *BReast CAncer susceptibility gene 2* (*BRCA2*) predispose to aggressive forms of prostate cancer which show poor response to taxane-based therapy, the standard treatment for castration-resistant, aggressive prostate cancer. Herein, we addressed the question whether changes in BRCA2 expression, a potential surrogate marker for BRCA2 activity, may affect the response of castration-resistant prostate cancer cells to 6-thioguanine (6-TG), a thiopurine used in the treatment of haematological malignancies. *Methods*: Yeast, normal prostate cells and castration-resistant prostate cancer cells were treated with 6-TG or its analogues, in presence or absence of paclitaxel, or with olaparib, a poly-(ADP-ribose) polymerase (PARP) inhibitor currently in clinical trials for treatment of metastatic castration-resistant prostate cancer, and cell proliferation, apoptosis and androgen receptor (AR) levels were measured. *Results*: 6-TG inhibited cell proliferation in yeast, normal and castration-resistant prostate cancer cells but promoted apoptosis only in cancer cells. Suppression of BRCA2 expression by siRNA or shRNA increased the sensitivity to 6-TG- and olaparib-induced apoptosis but did not affect cancer cell response to taxane. Intriguingly, 6-TG reduced AR expression levels independently on BRCA2 expression. Instead, olaparib decreased AR levels only in BRCA2-knockdown prostate cancer cells. Notably, overexpression of BRCA2 resulted in resistance of castration-resistant prostate cancer cells to 6-TG-, taxane- and olaparib-based treatment but promoted sensitivity to apoptosis induced by 2-amino-6-bromopurine and 2,6–dithiopurine, two 6-TG analogues. *Conclusions*: Our results provide a pre-clinical rationale for the use of 6-TG in the treatment of BRCA2-deficient castration-resistant prostate cancers, and of certain 6-TG analogues for treatment of BRCA2-proficient prostate cancers.

## 1. Introduction

Prostate cancer is the second leading cause of cancer death in the Western world. While the overall 5-year survival of patients with primary prostate cancer reaches 98%, it drops to 30% when the cancer progresses to a metastatic disease (www.cancer.org; American Cancer Society). Prostate cancer cells depend on androgens for their growth, thus androgen-deprivation therapy has been the mainstay for treatment of non-metastatic and early metastatic prostate cancer. However, all patients invariably progress during the course of their disease since prostate cancer cells adapt to the androgen-deprived environment becoming “castration-resistant”, which is considered the advanced stage of the disease [1]. Several mechanisms are implicated in prostate cancer progression to advanced stage [1]. Among them, deletion of the tumor suppressor Phosphatase and Tensin Homolog (PTEN) is frequently observed during cancer progression [2]. Amplification of the androgen receptor (AR) gene is considered one of the main mechanisms driving transition to a castration-resistant phenotype [3,4,5]. Current treatment for advanced prostate cancer relies on taxane-based therapy and on new generation antiandrogen drugs (abiraterone, enzalutamide) able to inhibit the activity of the AR [6,7]. Besides the AR, estrogens and their receptors have also been implicated in prostate cancer initiation and progression but the translation into a potential therapeutic strategy still remains challenging [8]. 

*BReast CAncer susceptibility gene 2* (*BRCA2*) is a tumor suppressor gene that when mutated confers increased sensitivity to several cancer types, including prostate carcinoma [9,10]. BRCA2 promotes repair of DNA double-strand breaks by homologous recombination (HR) [11]. We have previously reported that silencing of BRCA2 expression promotes resistance to anoikis, i.e., apoptosis induced by detachment of epithelial cells from the extracellular matrix [12], which is considered an important step in the metastatic cascade [13]. Notably, the mechanism involved in modulation of apoptosis by BRCA2 was found to be evolutionarily conserved in yeast and humans [12]. Prostate cancer patients with inherited BRCA2 mutations have a very aggressive disease, with higher grade, advanced stage at diagnosis and poor survival [14,15,16,17,18,19,20], making a priority the development of novel therapeutic strategies for these patients. In addition, metastases derived from castration-resistant prostate cancers, besides showing AR amplification, display also frequent somatic deletions of the *BRCA2* gene [4]. Several clinical trials are currently evaluating the use of targeted therapies such as poly-(ADP-ribose) polymerase (PARP) inhibitors in BRCA-associated prostate cancer [6,7]. PARP is a DNA-binding protein that promotes repair of DNA single-strand breaks through the base excision and repair (BER) pathway. Suppression of the base excision and repair pathway by PARP inhibitors in BRCA2-deficient/HR-defective cancer cells has a synthetically lethal effect, because it generates an overwhelming genomic instability that fosters cancer cells to death [21], thus making PARP inhibitors promising candidates for the treatment of BRCA2-deficient castration-resistant prostate cancers. However, BRCA2-mutated cancers often become resistant to PARP inhibitors via different mechanisms, including recovery of BRCA2 functionality due to secondary BRCA2 mutations [22,23].

The thiopurine 6-thioguanine (6-TG) is a chemotherapeutic drug that induces DNA damage and is successfully used in the treatment of childhood acute lymphoblastic leukemia and other forms of leukemias [24]. Previous studies on its efficacy in various solid tumors have shown infrequent but positive results and have suggested that combinations of 6-TG with other agents may enhance the antitumor effects [25,26]. Recently, 6-TG was found to be a potent inhibitor of ubiquitin-specific protease 2, which plays a critical role in prostate tumor cell survival [27]. Limited data are available on the effect of this drug on solid tumors also due to the toxicity that 6-TG may have on normal cells, this limiting its protracted use in therapy. So far, the potential antitumor effect of 6-TG has never been tested in castration-resistant prostate cancer cells. 

Yeast is a useful model organism for studying tumorigenic mechanisms [28] and for development of advanced technologies for drug discovery [29]. In particular, in BRCA2-expressing yeast cells, a high increase in both intra- and inter-recombination events occurs, and the expression of selected BRCA2 variants differentially affects yeast recombination [30], showing that BRCA2 function in homologous recombination-mediated DNA repair can be recapitulated in yeast. Thus, we first screened the effects of 6-TG and of its selected analogues on yeast cell growth and viability. We then investigated the effect of 6-TG alone and in combination with the taxane paclitaxel on normal immortalized and castration-resistant prostate cancer cells, and its dependence on BRCA2 expression. The effect of 6-TG treatment in BRCA2-knockdown prostate cancer cells before and after reconstitution of BRCA2 levels by ectopic expression was compared with treatment with olaparib, a Food and Drug Administration (FDA)-approved PARP inhibitor.

## 2. Results

### 2.1. Effect of 6-TG and Its Analogues on Yeast Cell Growth and Viability

**Figure 1:**
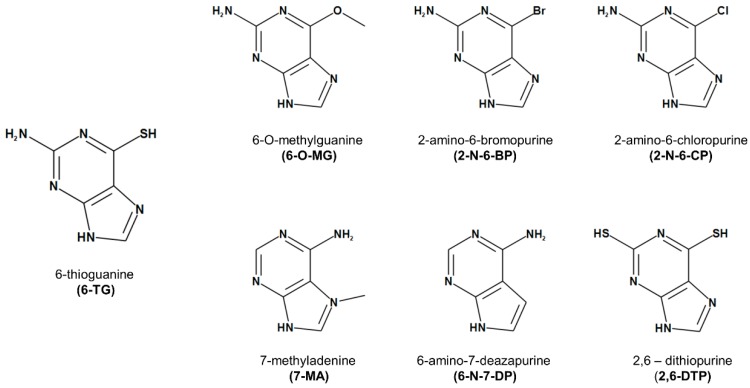
Chemical structure of 6-thioguanine and its analogues.

We first tested the effects on yeast cell growth of 6-TG and six of its analogues (Figure 1) in which either the thiol or the amino group is changed or lacking. 

**Figure 2:**
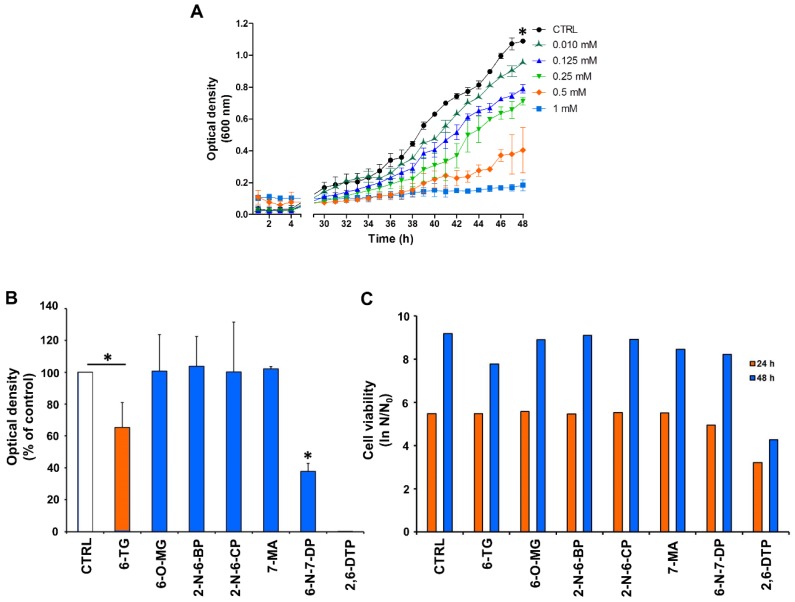
6-TG and its analogues 6-amino-7-deazapurine (6-N-7-DP) and 2,6-dithiopurine (2,6-DTP) impair cell growth of *Saccharomyces cerevisiae* yeast cells. (**A**) Yeast cells were treated with the indicated concentrations of 6-TG or with NaOH as control (black curve) and optical density was measured at 600 nm every hour up to 48 h. Each point represents the mean ± SD from cells of triplicate wells. Statistical significance difference with * *p* < 0.001, when comparing control with 1 mM, 0.5 mM, 0.25 mM and 0.125 mM, two-way ANOVA, Bonferroni post-hoc test. (**B**) Cell growth and viability in the presence of 6-TG and its analogues at 0.5 mM. Optical density at 48 hours was reported as percentage of control. The mean of three independent experiments ±SD was reported. Statistically significant difference with *, *p* < 0.05, when comparing control with 6-TG or 6-N-7-DP, and 6-TG with 6-N-7-DP, one-way ANOVA and Tukey’s multiple comparison post-hoc test. (**C**) Viability at 24 and 48 h of control and drug-treated cells was measured by counting colony forming units after two days of growth on Yeast Extract-Peptone-Dextrose (YPD) plates. N refers to the number of cfu at the indicated time, N_0_ refers to the number of cfu at time 0. Results from a typical experiment are shown.

A range of different concentrations of 6-TG, from 10 µM to 1 mM, was added to growing yeast cultures and optical density was measured. As reported in Figure 2A, yeast cell growth was sensitive to 6-TG in a dose-dependent manner. Forty-eight h after treatment with 0.5 and 1 mM 6-TG, the growth inhibition was 63% and 83%, respectively. Drug concentrations of 0.125 mM and 0.25 mM inhibited cell growth by 27% and 35%, respectively. 

Having established that 0.5 mM 6-TG partially but not completely inhibited yeast cell growth, we tested the effect of 6-TG analogues using this concentration. Yeast cell proliferation was not affected by 6-OMG, 2-N-6BP, 2-N-6CP and 7-MA (Figure 2B). 6-TG did inhibit cell growth by 35%. A significant growth reduction was observed in the presence of 6-N-7DP (62% inhibition with respect to control cells) and 2,6-DTP, the latter completely inhibiting cell proliferation (Figure 2B). Next, we tested cell viability by clonogenic assay after exposure to the drugs for 24 or 48 hours (Figure 2C). Only 2,6-DTP treatment resulted in decreased cell survival at 24 and 48 h (40% and 55% decrease). Overall, these results demonstrate that 6-TG, 6-N-7-DP and 2,6-DTP display an anti-proliferative effect in yeast, with 2,6-DTP significantly impairing cell viability.

### 2.2. Anti-Proliferative and Pro-Apoptotic Effect of 6-TG in Castration-Resistant Prostate Cancer Cells

**Figure 3:**
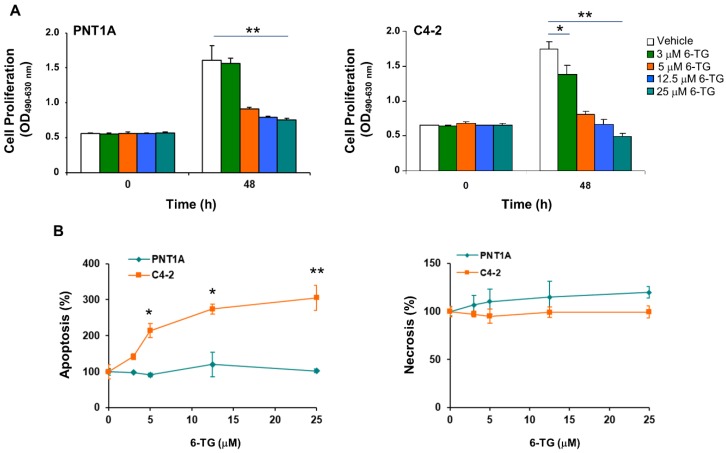
6-TG promotes apoptosis in cancer but not in normal prostate cells. (**A**,**B**) Prostatic Normal large T-antigen Immortalized 1A (PNT1A) and C4-2 cells were treated with different concentrations of 6-TG for 48 h. (**A**) Cell proliferation was measured by 2,3-bis-(2-methoxy-4-nitro-5-sulfophenyl)-2H-tetrazolium-5-carboxanilide (XTT) assay. Each point represents the mean ± SD from cells of triplicate wells. The experiment was repeated three times. (**B**) Cell apoptosis and necrosis were assessed by measuring mono- and oligo-nucleosomes in the cytosol and culture medium, respectively. Each point represents the mean ±SD from cells of triplicate wells. The experiment was repeated three times. * *p* < 0.001; ** *p* < 0.0001.

To investigate the effect of 6-TG in prostate cells, we used the PNT1A normal immortalized prostate cell line and the C4-2 and DU145 castration-resistant prostate cancer cells. C4-2 cells are an androgen-independent derivative of the androgen-dependent LNCaP prostate cancer cells derived from the left supraclavicular lymph node metastasis, which retain the AR’s T877A mutation present in the parental cell line (https://cancer.sanger.ac.uk/cosmic) [31]. The T877A mutation affects steroid binding and response to antiandrogens [31]. DU145 are androgen-unresponsive prostate cancer cells derived from a central nervous system metastasis. DU145 and C4-2 retain AR expression [32,33], both express the estrogen receptor β but not α [34,35], both express a mutant p53 (https://cancer.sanger.ac.uk/cosmic), and are PTEN-positive the first and PTEN-null the second, thus allowing to assess to potential contribution of PTEN status in the response to 6-TG. 6-TG treatment, up to 25 µM, exhibited anti-proliferative effects in both PNT1A and C4-2 cells (Figure 3A). Notably, about 45% of PNT1A cells and 55% of C4-2 cells survived 48 h after treatment with 5 µM or higher concentrations of 6-TG. C4-2 but not PNT1A cells underwent apoptosis after 6-TG treatment in a dose-dependent manner, reaching 214% ± 19 apoptosis at 5 µM, and 305.5% ± 34.7 at 25 µM 48 h after treatment (Figure 3B). Treatment with 6-TG did not induce necrosis up to 72 h at any of the tested concentrations (Figure 3B). 

**Figure 4:**
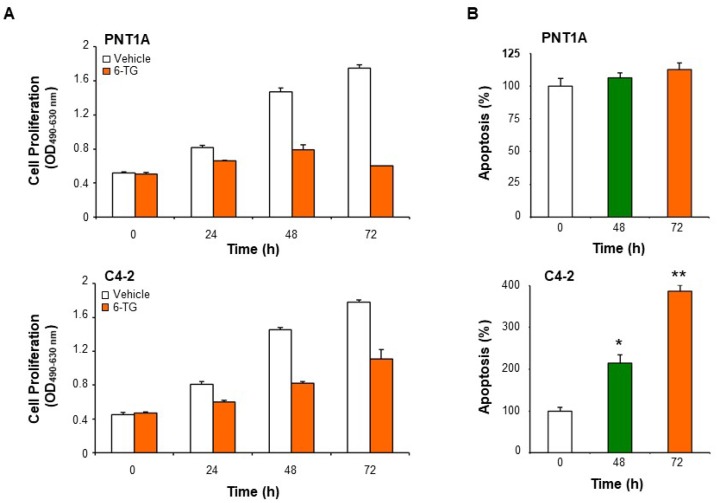
6-TG-mediated inhibition of cell proliferation is time-dependent. (**A**,**B**) PNT1A and C4-2 cells were treated with 5 µM 6-TG up to 72 h, and cell proliferation and apoptosis were measured by XTT (**A**) and Cell Death Detection (**B**) assay, respectively. Each point represents the mean ± SD from cells of triplicate wells. The experiment was repeated two times. * *p* < 0.001; ** *p* < 0.0001.

6-TG at a maximum concentration of 5 µM was used in all the following experiments. At 5 µM, the anti-proliferative effect of 6-TG was time-dependent both in PNT1A and C4-2 cells, with the strongest inhibitory effect observed at 72 h in PNT1A cells (Figure 4A). A time-dependent pro-apoptotic effect of 6-TG was detected in C4-2 but not PNT1A cells (Figure 4B).

### 2.3. BRCA2 Levels Affect the Sensitivity of Prostate Cancer Cells to 6-TG 

**Figure 5:**
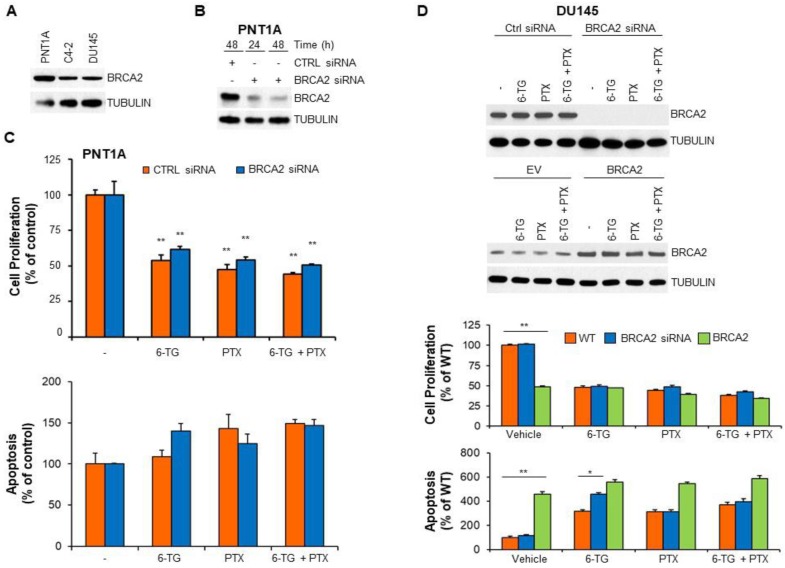
BReast CAncer susceptibility gene 2 (BRCA2) protein levels modulate the apoptotic cell response to 6-TG in prostate cancer cells. (**A**) Total protein extracts from PNT1A, C4-2 and DU145 cells were analyzed by immunoblotting using an antibody to BRCA2 or beta-tubulin, as loading control. (**B**,**C**) PNT1A cells were transiently transfected with siRNA to BRCA2 or non-targeting siRNA (CTRL) up to 48 h. (**B**) BRCA2 expression was analyzed by immunoblotting at 24 and 48 h after transfection with BRCA2 siRNA. (**C**) PNT1A cells were treated with solvent alone (−), 6-TG (5 µM), paclitaxel (10 nM) or a combination of the two drugs. Forty-eight hours after treatment, cell proliferation and apoptosis were measured by XTT (left panel) and Cell Death Detection (right panel) assay, respectively. Each point represents the mean ± SD from cells of triplicate wells. The experiment was repeated three times. (**D**) DU145 cells were transiently transfected with BRCA2 siRNA or a non-targeting siRNA (WT), and with recombinant BRCA2 cDNA or vector alone (EV). Twenty-four hours after transfection the cells were treated with solvent alone (−), 6-TG (5 µM) or paclitaxel (10 nM) or a combination of the two drugs. Forty-eight hours after treatment, cell proliferation and apoptosis were measured by XTT and Cell Death Detection assay (bottom panels) or the cells were lysed and total protein extracts analyzed by immunoblotting as indicated (upper panels). (**D**, bottom panels) Each point represents the mean ±SD from cells of triplicate wells. The experiment was repeated three times. * *p* < 0.001; ** *p* < 0.0001.

We have previously shown that BRCA2 downregulation promotes resistance to anoikis, a specialized apoptotic program initiated when epithelial cells detach from the extracellular matrix [12,36]. PNT1A normal prostate cells express higher BRCA2 levels than prostate cancer cells [37] (Figure 5A). To assess whether the apoptotic response to 6-TG treatment was affected by BRCA2 expression we transiently transfected PNT1A cells with BRCA2 siRNA (Figure 5B). Downregulation of BRCA2 expression did not affect PNT1A cell response either to 6-TG or to PTX or their combinations (Figure 5C).

**Figure 6:**
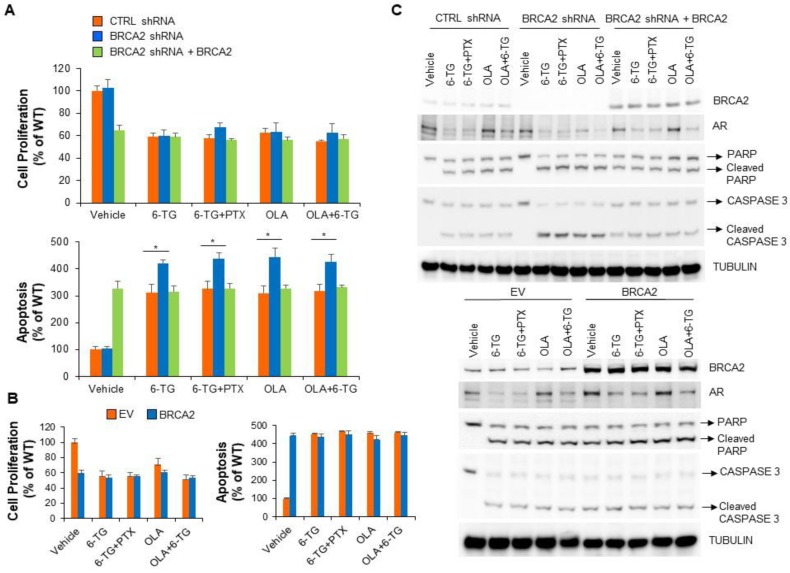
6-TG-induced apoptotic response in prostate cancer cells is similar in extent to the apoptotic effect induced by olaparib. (**A**,**B**) DU145 cells expressing doxycycline-inducible BRCA2 shRNAs or non-targeting shRNAs (WT) were treated with doxycycline (**A**). Thirty-six hours after doxycycline treatment, cells were added with solvent alone (Vehicle), 6-TG (5 µM), paclitaxel (10 nM) or a combination of the two drugs, and with olaparib (5 µM; OLA) or a combination of olaparib (5 µM; OLA) and 6-TG (5 µM). Where indicated, 24 h after doxycycline treatment and before addition of the drug/s, BRCA2 cDNA was transiently transfected in BRCA2 shRNA-expressing cells (BRCA2 shRNA+BRCA2). In **B**, DU145 cells were transiently transfected with BRCA2 cDNA or empty vector (EV) and added with the drugs 48 h after transfection. Forty-eight hours after drug treatment, cell proliferation and apoptosis were measured by XTT and Cell Death Detection assay (**A**,**B**) or the cells were lysed and total protein extracts analyzed by immunoblotting as indicated (**C**). In the graphs, each point represents the mean ± SD from cells of triplicate wells. The experiment was repeated two times. * *p* < 0.001.

BRCA2 knockdown by siRNA or shRNA in C4-2 and DU145 prostate cancer cells instead increased apoptosis induced by 6-TG or the PARP inhibitor olaparib but not by PTX (Figure 5D and Figure 6A,C and Supplementary Figure S1). Notably, downregulation of BRCA2 did not affect cell proliferation (Figure 5D and Figure 6A and Supplementary Figure S1). Transient overexpression of BRCA2 in prostate cancer cells resulted *per se’* in decreased cell proliferation, increased apoptosis and resistance to 6-TG, PTX or a combination of them (Figure 5D and Supplementary Figure S1). Similarly, rescue of BRCA2 levels in BRCA2 shRNA-expressing prostate cancer cells resulted in resistance to 6-TG, PTX and olaparib (Figure 6A–C). Of note, the effect of 6-TG on prostate cancer cell proliferation and apoptosis was comparable to that observed after olaparib treatment and a combination of olaparib with 6-TG didn’t increase the apoptotic response observed after single drug administration. Intriguingly, 6-TG treatment, alone or in combination with PTX or olaparib, reduced AR protein levels, independently on BRCA2 expression (Figure 6C). Instead, olaparib treatment reduced AR protein levels only in BRCA2-knockdown prostate cancer cells, and the addition of 6-TG had a synergic effect in reducing AR levels (Figure 6C). Taken together, these results demonstrate that BRCA2 levels modulate the sensitivity of prostate cancer cells to 6-TG to a similar extent as olaparib and that 6-TG treatment decreases the expression of AR independently on BRCA2. 

### 2.4. BRCA2 Expression Promotes Prostate Cancer Cell Sensitivity to 2-N-6-BP and 2,6-DTP 

**Figure 7:**
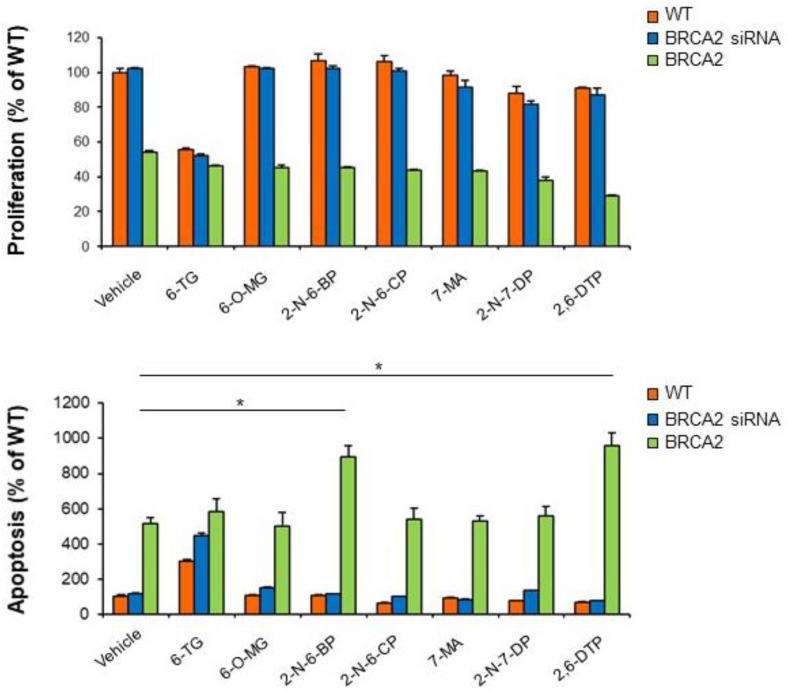
BRCA2 expression confers sensitivity to 2-N-6-BP and 2,6-DTP in castration-resistant prostate cancer cells. C-4 cells were transiently transfected with BRCA2 siRNA, a non-targeting siRNA (CTRL) or with recombinant BRCA2 cDNA. Twenty-four hours after transfection the cells were treated with 5 µM 6-TG, 6-O-MG, 2-N-6-BP, 2-N-6-CP, 7-MA, 2-N-7-DP, 2,6-DTP or solvent alone (−). Forty-eight hours after treatment, cell proliferation and apoptosis were measured by XTT (top panel) and Cell Death Detection (bottom panel) assay, respectively. Each point represents the mean ± SD from cells of triplicate wells. The experiment was repeated two times. * *p* < 0.01.

Since BRCA2 expression conferred resistance to 6-TG-induced apoptosis in castration-resistant prostate cancer cells, we then tested whether 6-TG analogues may have an effect on BRCA2-proficient prostate cancer cells. None of the 6-TG analogues tested did affect cell proliferation or apoptosis in wild-type or BRCA2-depleted castration-resistant cancer cells (Figure 7). 

Notably, overexpression of BRCA2 abolished any effects on prostate cancer cell proliferation in response to any compound tested and sensitized PC cells to apoptosis following treatment with 2-N-6-BP and 2,6-DTP (174% ± 12 and 187% ± 13, respectively, compared to untreated cells; *p* < 0.01). These results show that reconstitution of BRCA2 expression may render castration-resistant prostate cancer cells sensitive to certain 6-TG analogues.

## 3. Discussion

We report here that castration-resistant prostate cancer cells defective in BRCA2 are hypersensitive to 6-TG at levels comparable to olaparib treatment, and that this could be reversed by transient transfection of a vector expressing BRCA2. These results are consistent with a previous study showing that cancer cells, in which *BRCA2* is silenced by shRNA, become sensitive to a compound structurally related to 6-TG and to 6-TG itself [38]. Notably, yeast cells, that do not possess direct homologues of *BRCA2* [28], show sensitivity to 6-TG, suggesting that the mechanism of action of 6-TG is evolutionary conserved in BRCA2-lacking cells. 

We show that 6-TG inhibits cell proliferation both in normal and in cancer prostate cells but it promotes cell death by apoptosis only in cancer cells, suggesting a cancer-specific mechanism of 6-TG-induced apoptosis. We didn’t observe any difference in response to 6-TG between C4-2 (PTEN-null) and DU145 (PTEN-positive) prostate cancer cells, suggesting that the response is independent on PTEN status. Homologous recombination (HR) is implicated in the repair of 6-TG-induced double-strand DNA breaks (DSBs) [38]. Since the castration-resistant prostate cancer cells assayed in this study have decreased BRCA2 levels compared to the normal PNT1A cells, it is possible that reduced repair of 6-TG-induced DSBs by HR contributes to activation of apoptosis in cancer cells. In addition, 6-TG-mediated toxicity in cancer cells has been also associated with mitochondrial-dependent overproduction of ROS [39]. A previous study has shown that prostate cancer cells have higher ROS production compared with normal prostate cells through activation of the extramitochondrial source of ROS generator, NAD(P)H oxidase [40]. It’s plausible that prostate cancer cells, having already high basal levels of ROS, would not be able to cope with the detrimental excess of ROS generated by 6-TG treatment, thus undergoing apoptosis. The enzyme methylthioadenosine phosphorylase (MTAP) plays a major role in the metabolism of polyamines and its deficiency has been implicated in the response of cancer cells to 6-TG. MTAP is frequently deleted in several cancers [41]. A treatment strategy combining methylthioadenosine (MTA) with 6-TG has been proven successful in selectively killing MTAP-deficient tumor cells. In normal MTAP-proficient cells, MTA is cleaved by MTAP to 5-methylthioribose1-phosphate and to adenine. Adenine is phosphoribosylated by adenine phosphoribosyltransferase to form AMP, thus competitively inhibiting phosphoribosylation of 6-TG to a toxic nucleotide, and protecting normal cells while killing MTAP-deficient tumor cells [41]. However, MTAP is very rarely mutated in prostate cancer as it plays an essential role in the homeostatic regulation of prostate cells’ metabolite pools [42]. The castration-resistant prostate cancer cells used in this study, C4-2 and DU145, are MTAP-proficient [42], thus the differential toxicity of 6-TG in cancer *versus* normal prostate cells cannot be ascribed to a deficiency of MTAP in prostate cancer cells. Of note, we show that depletion of BRCA2, while increasing sensitivity to 6-TG in prostate cancer cells, did not affect the response in normal prostate cells, thus implying that other factors, together with BRCA2 levels, are important for modulating the sensitivity to 6-TG specifically in cancer cells. Notably, BRCA2 has been reported to protect against oxidative stress [43]. The constitutively higher levels of ROS in cancer cells compared with normal cells may be one of the factor cooperating with BRCA2 signaling in promoting 6-TG-mediated apoptosis in cancer cells. 

Our results show that treatment of castration-resistant prostate cancer cells with 6-TG significantly decreased AR levels, independently on BRCA2 expression. These findings uncover the potential role of 6-TG in promoting cancer cell death by downregulating AR signaling, suggesting that 6-TG may function as a “novel” antiandrogen drug for prostate cancer. Olaparib treatment reduced AR levels only in BRCA2-knockdown prostate cancer cells, indicating that 6-TG and olaparib regulate AR through different molecular mechanisms, the first being BRCA2-independent and the second BRCA2-dependent. Future investigations using AR rescue experiments are warranted to assess the role of 6-TG- and olaparib-mediated regulation of AR expression in the induction of apoptosis in castration-resistant prostate cancer cells. 

We show here that gain of BRCA2 expression in castration-resistant prostate cancer cells confers resistance to 6-TG- and olaparib-induced apoptosis but sensitivity to two 6-TG analogues, namely 2-N-6-BP and 2,6-DTP. Notably, normal prostate epithelial cells (expressing high levels of BRCA2) and BRCA2-defective castration-resistant prostate cancer cells are resistant to the two analogues, indicating that the mechanism involved in 2-N-6-BP- and 2,6-DTP-mediated apoptosis is effective only in cancer cells and requires BRCA2. Issaeva et al. [38] have previously shown that BRCA2-defective cancer cells, including the Capan-1 pancreatic cancer cell line, retain sensitivity to 6-TG after *BRCA2* genetic reversion. The discordance with our results may be due to the different cancer type as well as to the different genetic background. Indeed, Capan-1 are MTAP-deficient [44], thus explaining their retained sensitivity to 6-TG after restoring BRCA2 expression. BRCA2 reversion conferred resistance of castration-resistant prostate cancer cells to olaparib, consistently with previous studies in breast and prostate cancer patients showing acquired resistance to olaparib in BRCA2-associated cancers due to *BRCA2* reversion mutations [45,46,47]. Intriguingly, we show that BRCA2-proficient castration-resistant cancer cells become resistant to paclitaxel treatment, suggesting that *BRCA2* genetic reversion or high basal levels of BRCA2 in prostate cancer patients may predict resistance to taxane-based therapy. Future in vivo experiments in mouse models will assess the therapeutic potential of 6-TG in the treatment of BRCA2-deficient prostate cancers, and of 2-N-6-BP- and 2,6-DTP in the treatment of BRCA2-proficient, advanced prostate cancers. 

In this context the comparison of results obtained with human and yeast cells is of interest. 6-TG is a non-competitive inhibitor of human ubiquitin-specific protease 2 (USP2) [27], a member of the largest class of deubiquitinating (DUB) enzymes in yeast and humans playing an essential role in numerous cellular processes including cell cycle regulation and DNA repair [48]. Allosteric binding of 6-TG to the enzyme involves covalent bonding interaction of its thiol group with a specific Cys and polar interaction of its amino group with a specific Gln residue, leading to the movement of a conserved Asp residue playing an essential role in catalysis [27]. Since the USP domain fold is highly conserved despite low sequence similarity (for refs see [27]), it is tempting to speculate that 2,6-DTP containing two thiols can inhibit a DUB enzyme conserved in yeast and humans, whereas 2-N-6-BP may exert its effect only on human enzymes. Indeed, both 6-TG analogues induce cell death in cells with an efficient DNA repair system. With this respect, it should be noted that Usp11 has been described as a DUB that exhibits pro-survival functions as part of the cellular response to DNA damage within the BRCA2 pathway, even though independently of BRCA2 deubiquitination [49].

Overall, our study provides a pre-clinical rationale for further studies aimed at evaluating the use of 2-N-6-BP and 2,6-DTP for the treatment of BRCA2-proficient castration-resistant prostate cancers and suggests that 6-TG and the PARP inhibitor olaparib may have similar therapeutic potential in the treatment of BRCA2-deficient advanced prostate cancers.

## 4. Materials and Methods

### 4.1. Chemicals and Antibodies

The following chemicals were used in this study: 6-thioguanine (6-TG, Cat. # A4882), 6-O-methylguanine (6-O-MG; Cat. # 363057), 2-amino-6-bromopurine (2-N-6-BP; Cat. # 475254), 2-amino-6-chloropurine (2-N-6-CP; Cat. # 342300), 7-methyladenine (7-MA; Cat. # 666548), 6-amino-7-deazapurine (6-N-7-DP; Cat. # 722332), 2,6-dithiopurine (2,6-DTP; Cat. # sc-256365), Paclitaxel (PTX; Cat. # T1912). The chemical structure of 6-TG and the analogs tested in this study are reported in Figure 1. All chemicals were purchased from Sigma Aldrich (Milan, Italy), except for 2,6-DTP which was purchased from Santa Cruz Biotechnology (Santa Cruz, CA, USA). Stock solutions of 6-TG and its analogues were prepared in 0.4 M NaOH for yeast experiments, and in PBS for studies involving human cells; PTX was dissolved in DMSO. Antibody to BRCA2 (H-300) was from Santa Cruz Biotechnologies. Antibodies to caspase 3 and PARP were from Cell Signaling Technologies (Danvers, MA, USA). Monoclonal antibody to AR was from Abcam (Cambridge, MA, USA). Anti-tubulin monoclonal antibody was from Sigma. 

### 4.2. Cell Lines and Yeast Cell Cultures

PNT1A human normal immortalized prostate cells were obtained from ECACC (European Collection of Cell Cultures, Salisbury, UK). C4-2 human prostate carcinoma cell line was a kind gift of Dr. J.T. Hsieh (UT Southwestern Medical Center, Dallas, TX, USA). DU145 human prostate carcinoma cell line was obtained from ATCC. Human cell lines were grown in RPMI-1640 medium supplemented with 10% fetal bovine serum and 1 mM sodium pyruvate. 

The drug-sensitive *Saccharomyces cerevisiae* yeast strain AD1-8-TAX (genotype: MATα, PDR1-3, his1, Δyor1::hisG, Δsnq2::hisG, Δpdr5::hisG, Δpdr10::hisG, Δpdr11::hisG, Δycf1::hisG, Δpdr3::hisG, Δpdr15::hisG, leu2Δ1, trpΔ63, his4-917, URA3/ura3-52, tub2-His6-A19K-T23VG26D-N227H-Y270F) was kindly provided by Prof. M. Gupta (Iowa State University, Ames, IA, USA). This strain has a decreased level of the ABC multidrug transporters, is paclitaxel-sensitive carrying mutations in the beta-tubulin gene, and has been transformed with the episomal plasmid YEplac195 with a URA3 marker for further studies in an heterologous expression setting [50]. Yeast cells were pre-grown overnight with constant orbital shacking (150 rpm) at 30 °C in SC-GEG medium (Synthetic Complete 3% glycerol, 0.5% ethanol, 0.5% glucose without uracil) and then transferred in SC-Gal medium (Synthetic Complete −2% Galactose) at 26 °C. For viability assays, cells were grown on rich YPD medium (1% Yeast Extract, 2% Peptone, 2% Dextrose, 2% agar).

### 4.3. Cell Cytotoxicity Tests

#### 4.3.1. Cell Proliferation and Cell Death Assay

Human prostate cells (5.000 cells/well) were plated in 96-well plates. After 24 h, fresh medium containing 5 µM 6-TG, 5 µM 6-TG analogues or 5 µM olaparib was added to the cells. In some experiments, cells were added with 100 nM PTX. Cell proliferation was measured using the Cell Proliferation Kit II (XTT; Roche, Milan, Italy). Apoptosis (cytoplasmic mono- and oligo-nucleosomes) and necrosis were measured using the Cell Death Detection ELISA^PLUS^ kit (Roche, Milan, Italy) according to the manufacturer’s instructions. 

#### 4.3.2. Yeast Growth and Cell Viability Assays

Cytotoxicity assays in yeast cells were performed by monitoring cell growth or cell viability in liquid cultures. In particular, the yeast growth curves in the presence of 6-TG were obtained by means of a Bioscreen C MBR instrument (Bioscreen, Carlo Erba Reagents S.r.l., Cornaredo-Milan, Italy). Each well of the Bioscreen 100-well Honeycomb plate was filled with 2.000 cells/150 μl of SC-Gal medium. The optical density was recorded at 600 nm every hour for 48 hours. 

For the drug screening assay, an appropriate volume of the pre-culture was diluted in SC-Gal medium to obtain a final concentration of 1.6 × 10^4^ cells/mL. The culture was incubated at 26 °C under shaking (150 rpm), in the presence of 500 µM 6-TG or its analogues. After 48 h, the culture’s optical density was measured at 600 nm. To assess cell viability in a clonogenic assay, yeast cells were plated on rich YPD medium (1% Yeast Extract, 2% Peptone, 2% Dextrose, 2% agar) before (time 0) and during drug treatment and the number of colony forming units (cfu) was determined. The logarithm of the number of cfu at the indicated time *versus* the number of cfu at time 0 (ln N/N0) is reported as a measure of cell viability. 

### 4.4. Immunoblotting

Cells at 60–70% confluence were lysed in lysis buffer containing 10 mM Tris-HCl (pH 7.5), 150 mM NaCl, 0.3 % Triton-X-100, protease inhibitor cocktail (Roche, Indianapolis, Indiana, USA), and phosphatase inhibitor cocktail (Thermo Fisher Scientific, Waltham, MA, USA). Total protein extracts were analyzed by immunoblotting as described previously [12]. 

### 4.5. Transient and Stable Transfection

Cells were transiently transfected with BRCA2 cDNA or a specific BRCA2 siRNA as previously described [51]. DU145 cells were stably transfected with doxycycline-inducible BRCA2 shRNAs targeting BRCA2 3′-UTR (Dharmacon, Lafayette, CO, USA). Where indicated, 24 h after doxycycline treatment and before addition of the drug/s, BRCA2 shRNA-expressing cells were transiently transfected with a plasmid encoding BRCA2 cDNA.

### 4.6. Statistical Analysis 

Data are expressed as the mean ±SD. Comparisons to evaluate statistical significance among samples were performed using ANOVA test and Bonferroni or Tukey post hoc test, as specified in the text. 

## 5. Conclusions

Taken together, our findings show that the FDA-approved 6-TG may represent a promising therapeutic drug for treatment of BRCA2-deficient castration-resistant prostate cancers. Our results provide also the first pre-clinical evidence that certain 6-TG analogues, namely 2-N-6-BP and 2,6-DTP, may be effective for treatment of BRCA2-proficient cancers.

## Supplementary Materials

The following are available online at https://www.mdpi.com/2072-6694/11/7/945/s1, Figure S1: BRCA2 protein levels modulate the apoptotic cell response to 6-TG in prostate cancer cells.
