# Supplementary figures and images for: 6-Thioguanine and Its Analogs Promote Apoptosis of Castration-Resistant Prostate Cancer Cells in a BRCA2-Dependent Manner
Source: Cancers (Basel). 2019 Jul 5;11(7):945. doi: 10.3390/cancers11070945 (PMC6678799; doi:10.3390/cancers11070945)

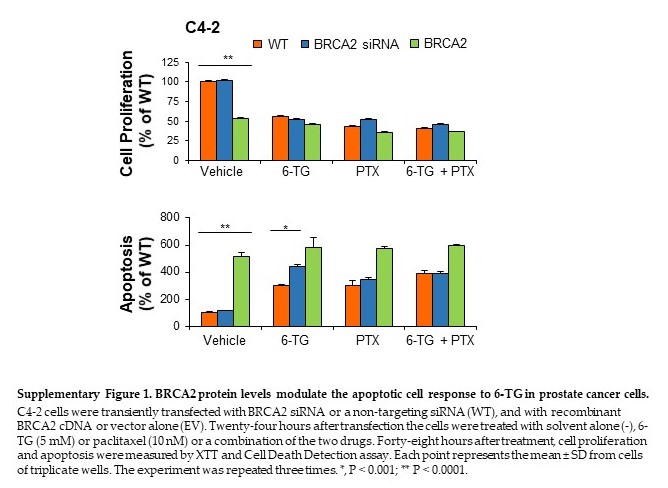

Supplement: Supplementary file 1 [file cancers-11-00945-s001.zip › cancers-544365-supplementary.JPG]
